# Pattern and factors associated with congenital anomalies among young infants admitted at Bugando medical centre, Mwanza, Tanzania

**DOI:** 10.1186/1756-0500-7-195

**Published:** 2014-03-29

**Authors:** Florentina Mashuda, Antke Zuechner, Phillipo L Chalya, Benson R Kidenya, Mange Manyama

**Affiliations:** 1 Department of Pediatrics and Child Health, Catholic University of Health and Allied Sciences, Mwanza, Tanzania; 2 Department of Pediatrics and Child Health, Bugando Medical Centre, Mwanza, Tanzania; 3 Department of Surgery, Bugando Medical Centre, Mwanza, Tanzania; 4 Department of Biochemistry and Molecular Biology, Catholic University of Health and Allied Sciences, Mwanza, Tanzania; 5 Department of Anatomy and Cell Biology, Catholic University of Health and Allied Sciences, Mwanza, Tanzania

## Abstract

**Background:**

Congenital anomalies or birth defects are among the leading causes of infant mortality and morbidity around the world. The impact of congenital anomalies is particularly severe in middle- and low-income countries where health care resources are limited. The prevalence of congenital anomalies varies in different parts of the world, which could reflect different aetiological factors in different geographical regions.

**Methods:**

Between October 2012 and January 2013, a cross-sectional study was conducted involving young infants below 2 months of age, admitted at a university teaching hospital in Tanzania. Face-to-face interviews with parents/caretakers of young infants were carried out to collect socio-demographic and clinical information. Physical examinations were performed on all young infants. Echocardiography, X-ray, cranial as well as abdominal ultrasonographies were performed when indicated.

**Results:**

Analysis of the data showed that among 445 young infants enrolled in the study, the prevalence of congenital anomalies was 29%, with the Central Nervous System (CNS) as the most commonly affected organ system. Maternal factors that were significantly associated with congenital anomalies included the lack of peri-conceptional use of folic acid (OR = 3.1; 95% CI = 1.4-6.7; p = 0.005), a maternal age of above 35 years (OR = 2.2; 95% CI = 1.1-4.3; p = 0.024) and an inadequate attendance to antenatal clinic (OR = 2.1; 95% CI = 1.4-3.3; p < 0.001). Infant factors that were significantly associated with congenital anomalies were female sex, a birth weight of 2.5 kg or more, singleton pregnancy and a birth order above 4.

**Conclusions:**

Due to the high prevalence of congenital anomalies observed in this particular context, the hospital should mobilize additional resources for an optimal and timely management of the patients with congenital anomalies. In this study, the proportion of women taking folic acid supplements during early pregnancy was very low. Efforts should be made to ensure that more women use folic acid during the peri-conceptional period, as the use of folic acid supplement has been linked by several authors to a reduced occurrence of some congenital anomalies.

## Background

Congenital anomalies or birth defects are structural or functional anomalies, including metabolic disorders, which are present at the time of birth. Some of the congenital anomalies may be life threatening, may impair function or interfere with the cosmetic value of an individual, hence an immediate management is required. The long-term disability caused by congenital anomalies may have a significant impact not only on a child’s well being and development, but also on families, health care systems and societies [1]. The impact of congenital anomalies is severe in middle- and low-income countries. As a matter of fact, it is estimated that approximately 95% of the children who die from birth defects are from those countries [1].

Worldwide, the incidence of congenital anomalies varies between geographical regions but it is estimated that 3-7% of children are born with birth defects [1-3]. Approximately, 270,000 newborns die during the first 28 days of life every year from congenital anomalies [4]. In the United States of America, congenital anomalies reportedly affect 2-5% of all live births [3]. The magnitude of congenital anomalies in Asia has been shown to vary with reported incidences of 2.5% in India and 1.3% in China [5,6]. In the Middle East, where consanguineous marriages are common, the prevalence of major congenital anomalies is reported to be 2–2.5%, the highest prevalence (7%) being found in consanguineous marriages [1,5,7]. In Africa, some of the rare studies on congenital anomalies have reported an incidence between 1.5% and 2.5% in Egypt and East Africa (Kenya and Uganda) respectively [8-10]. Reports on the incidence of congenital anomalies in the developing world should be taken with caution, as the absence of birth defect registries in most of these countries, the deficiency in diagnostic capabilities and unreliable medical records and health statistics might increase the chances of underestimation. Most of the studies done in Africa have been retrospective hospital-based studies which are usually affected by underreporting and other sources of ascertainment bias [11].

About 60% of the causes of congenital anomalies in humans is still unknown [12]. However, in about 25% of congenital anomalies, the causes seem to be “multifactorial”, indicating a complex interaction between genetic and environmental risk factors [12]. A wide range of environmental risk factors have been associated with the occurrence of congenital anomalies [12]. Exposure during pregnancy to drugs such as thalidomide and phenytoin, alcohol, cigarette smoking, certain environmental chemicals and high doses of radiation have all been implicated in the causation of congenital anomalies [13-15]. The occurrence of congenital anomalies has also been associated with advanced maternal and paternal age, parental consanguinity, increasing birth order and low birth weight [14,16].

The pattern of congenital anomalies varies from region to region and also over time [2]. Generally, congenital anomalies that involve the CNSand the cardiovascular and musculoskeletal systems have been reported to be the most common [4,12,17].

Epidemiological surveys of congenital anomalies in various parts of the world with different environment, socioeconomic status are likely to give out vital information on the prevalence, pattern and risk factors for congenital anomalies in different areas [18]. The current study was conducted in order to determine the prevalence, pattern and factors associated with congenital anomalies among young infants admitted at a university hospital (Bugando Medical Centre), Mwanza, Tanzania. Tanzania is a low-resource African country where the magnitude of congenital anomalies and the associated factors are not well documented. Results from this study will support the development of strategies for improving the management and rehabilitation of patients with congenital anomalies in this particular context. Information on associated factors may shed light on their roles as risk factors for the occurrence of congenital anomalies hence providing baseline data for future studies and public health measures.

## Methods

### Study setting and patients recruitment

This was a cross-sectional hospital-based study involving young infants below 2 months of age admitted at the Bugando Medical Centre between October 2012 and January 2013. The Bugando Medical Centre is a 1000-bed capacity referral hospital in Tanzania serving about 15 million people. The hospital handles most of the congenital anomaly cases of the regions around Lake Victoria because of its capacity to offer specialized management. We enrolled young infants admitted at neonatal wards, pediatrics surgical ward, general pediatrics wards and pediatrics semi-intensive/critical care units. The ethical approval was obtained from the joint Catholic University of Health and Allied Sciences (CUHAS)/BMC ethical review board. The sample size was calculated using the Yamane Taro formula [19], whereby a total of 445 young infants with and without congenital anomalies were recruited in the study. Patients were recruited serially until the desired sample size was reached. After a written consent was obtained from the parents/caretakers, face-to-face interviews with parents/caretakers of young infants were carried out to collect socio-demographic and clinical information, such as maternal age and parity, history of Diabetes Mellitus, drug intake, exposure to X-ray, history of congenital malformation in the family, parental consanguinity, residential area, maternal exposure to pollutants and number of antenatal clinic visits. The birth weight of young infants was obtained by asking the mother/caretaker and then confirmed by observation of the antenatal card, infants Reproductive and Child Health number one (RCH1) card or other hospital documents, e.g. patient files.

### Physical examination and imaging investigations

All young infants had a thorough physical examination (general and systemic) performed by the paediatrician. Echocardiography, X-ray imaging, cranial and abdominal ultrasonography were performed when required. X-ray films were interpreted by two independent radiologists. Ultrasonography was performed by the radiologists and the senior sonographer. Echocardiography was executed by a paediatrician experienced in paediatric echocardiography. Due to lack of relevant equipment and qualified staff, genetic screening could not be performed.

The patterns of congenital anomalies were classified according to the International Statistical Classification of Diseases and Related Health Problems 10th Revision (ICD-10) Version for congenital malformations, deformations and chromosomal abnormalities [20]. Young infants with multiple congenital anomalies were grouped depending on whether those anomalies qualified as a specific syndrome or not. If they qualified as a specific syndrome, they were then categorized into that syndrome. If no syndrome could be classified by those anomalies and two systems were involved, both systems were recorded. When more than two systems were involved, it was recorded as multiple congenital anomalies.

### Data management and statistical analysis

Data were managed using EpiData version 3.1 (Atlanta, US) and analysis was done using STATA version 11 (College Station, Texas). Categorical variables were summarized as proportions and were compared using Pearson’s Chi square test while continuous data was described as medians (interquartile range). Univariate followed by multivariate logistic regression analyses were applied to determine the factors associated with congenital anomalies. Factors with a p < 0.1 on univariate analysis were subjected to multivariate logistic regression analysis. Crude (unadjusted) and adjusted odds ratios were calculated to quantify the strength of association between the factors and congenital anomalies. The 95% confidence intervals were determined and the factors with a p-value of less than 0.05 were considered to have a significant association with congenital anomalies.

## Results

Among the 445 young infants admitted at BMC, 243 (54.6%) were males, 200 (44.9%) were females and 2 (0.5%) had ambiguous genitalia. The age range was between one day and 60 days, with a median age of 4 with an inter-quartile range of 2–8 days. Singleton and twins accounted for 396 (89%) and 49 (11%) respectively, while a birth order of equal or less than four accounted for 367 (83%). A family history of congenital anomalies was found among 75 (3%) infants of the study sample.

Parental demographics showed that 339 (76.7%) of the mothers of young infants were aged between 20 and 35 years of age and only 67 (15%) had used folic acid supplements during the first trimester of last pregnancy. Fifty mothers (11%) were exposed to passive smoking, 12 (3%) used alcohol during pregnancy and none had a history of active smoking or exposure to X-ray irradiation during pregnancy. There was no history of consanguinity and only one mother was using antiretroviral drugs during her pregnancy.

**Figure 1:**
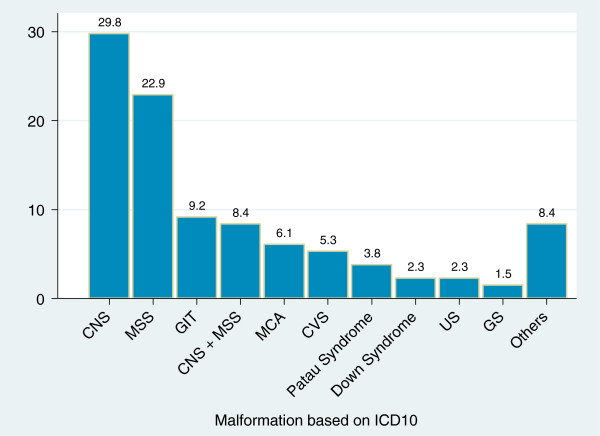
**Distribution of congenital anomalies according to ICD 10 among young infants admitted at Bugando Medical Centre from October 2012 to Jan 2013. Key: CNS:** central nervous system, **MSS:** musculoskeletal system,** GIT:** gastrointestinal tract,** MCA:** multiple congenital anomalies involving at least 3 systems,** CVS:** cardiovascular system,** US:** urinary system,** GS:** genital system. **Others:** CNS + GIT, CVS + GIT, GIT + GS, GIT + MSS, MSS + CVS, Pierre Robbin’s syndrome, Skin, Skin + MSS.

During the study period, 131 infants among the 445 examined were found with congenital anomalies. This gives a prevalence rate of 29%. The most affected body system was the central nervous system which accounted for 39 of the cases (29.8%), followed by the musculoskeletal and gastrointestinal systems for 30 (22.9%) and 12 (9.2%) cases respectively (Figure 1). Among the infants with CNS malformations, spina bifida was the most common, followed closely by hydrocephalus, with 16 (38.5%) and 14 (35.9%) cases respectively (Table 1). Other CNS congenital anomalies are shown in Table 1.

**Table 1:** Specific CNS congenital anomalies among young infants with CNS anomalies

**CNS anomalies**	**Number (%)**
Spina bifida	16 (41.1)
Spina bifida and hydrocephalus	1 (2.6)
Abnormal brain tissue	1 (2.6)
Encephalocele and microcephaly	5 (12.8)
Hydrocephalus	14 (35.9)
Hydrocephalus with Dandy Walker cyst	1 (2.6)
Meningoencephalocele	1 (2.6)
**Total**	**39 (100)**

Omphalocele was found to be the commonest congenital anomaly affecting the musculoskeletal system, accounting for 8 (26.8%) of all cases with musculoskeletal anomalies, followed by gastroschisis 6 (20.0%) and polydactyl 5 (16.7%) [Table 2]. The most common anomaly of the gastrointestinal tract (GIT) was imperforate anus, accounting for 7 (58%) of all GIT malformations (Table 2). Other malformations of the musculoskeletal and GIT are shown in Table 2.

**Table 2:** Musculoskeletal and gastrointestinal tract anomalies among young infants

**Musculoskeletal system anomalies**	**Number (%)**
Gastroschisis	6 (20.0)
Omphalocele	8 (26.7)
Polydactyly	5 (16.7)
Talipes	4 (13.3)
**Gastrointestinal tract anomalies**	
Imperforate anus	7 (58.3)
Oesophageal atresia	2 (16.7)
Ankyloglossia (tongue tie)	2 (16.7)
Cleft lip and palate	1 (8.3)

Maternal factors that were associated with congenital anomalies were: Non use of folic acid during pregnancy (OR = 3.1; 95% CI = 1.4-6.7; p = 0.005), a maternal age above 35 years (OR = 2.2; 95% CI = 1.1-4.3; p = 0.024), and three or less antenatal clinic (ANC) visits (OR = 2.1; 95% CI = 1.4-3.3; p < 0.001) [Table 3]. Infant factors that were significantly associated with congenital anomalies were: Female sex (OR = 1.8; 95% CI = 1.1-2.8; p = 0.013), a birth weight of 2.5 kg or more (OR = 2.3; 95% CI = 1.4-3.9; p = 0.002), singleton pregnancy (OR = 3.5; 95% CI = 1.2-10.9; p = 0.027) and a birth order of 4 and above (OR = 4.4; 95% CI = 2.6-7.6; p < 0.001) [Table 4].

**Table 3:** Univariate and multivariate analysis for parental factors associated with congenital anomalies among young infants admitted at Bugando Medical Centre

**Parental risk factor**	**Congenital malformations**		**Unadjusted**		**Adjusted**	
	**Yes**	**No**	**OR [95% CI]**	**P-value**	**OR [95% CI]**	**P-value**
*Maternal age*						
≤35	112 (27.6)	293 (72.4)	1		1	
>35	19 (47.5)	21 (52.5)	2.4 [1.2-4.6]	0.010	2.2 [1.1-4.3]	0.024
*Antenatal visit*						
>3 visit	51 (21.3)	188 (78.7)	1		1	
≤3 visit	79 (38.9)	124 (61.1)	2.3 [1.5-3.6]	<0.001	2.1 [1.4-3.3]	<0.001
*Passive smoking*						
No	115 (29.3)	277 (70.7)	1			
Yes	15 (30)	35 (70)	1.03 [0.5-2]	0.923	_	_
*Maternal alcohol intake*						
No	129 (30)	301 (70)	1			
Yes	1 (8.3)	11 (91.7)	0.21 [0.3-1.7]	0.140	_	_
*Maternal use of folic acid*						
Yes	8 (11.9)	59 (88.11)	1		1	
No	122 (32.5)	253 (67.5)	3.6 [1.6-7.7]	0.001	3.1 [1.4-6.7]	0.005
*Paternal age*						
<45	117 (28.5)	293 (71.5)	1			
≥45	13 (40.6)	19 (59.4)	1.7 [0.8-3.6]	0.152	_	_

**Table 4:** Univariate and multivariate analysis for infant factors associated with congenital anomalies among young infants admitted at Bugando Medical Centre

**Risk factors**	**Congenital malformations**		**Unadjusted**		**Adjusted**	
	**Yes n (%)**	**No n (%)**	**OR [95% CI]**	**P-value**	**OR [95% CI]**	**P-value**
*Sex*						
Male	64 (26.3)	179 (73.7)	1		1	
Female	65 (32.5)	135 (67.5)	1.3 [0.9-2.0]	0.156	1.8 [1.1-2.8]	0.013
*Birth order*						
≤4	87 (23.7)	280 (76.3)	1		1	
>4	43 (57.3)	32 (42.7)	4.3 [2.6-7.3]	<0.001	4.4 [2.6-7.6]	<0.001
*Pregnancy type*						
Twin	4 (8.2)	45 (91.8)	1		1	
Singleton	127 (32.1)	269 (67.9)	5.3 [1.9-15.1]	0.002	3.5 [1.2-10.9]	0.027
*History of birth defect*						
No	125 (29.1)	304 (70.9)	1			
Yes	5 (38.5)	8 (61.5)	1.5 [0.5-4.7]	0.470	-	-
Birth weight						
<2.5 kg	26 (16.1)	135 (83.9)	1		1	
≥2.5 kg	105 (37.0)	179 (63.0)	3.0 [1.9-4.9]	<0.001	2.3 [1.4-3.9]	0.002

## Discussion

Congenital anomalies are among the major causes of childhood morbidity and mortality in many countries around the world. The objective of this cross-sectional study was to report on the prevalence, pattern as well as factors associated with congenital anomalies among young infants admitted at the Bugando Medical Centre in Mwanza, Tanzania. Due to our study design, it was not possible to determine a causal association of these factors with congenital anomalies. In addition, the limited investigative ability at BMC, made it impossible to perform genetic/chromosomal or metabolic disorders studies.

About 29% of all young infants admitted at BMC during the study period were found to have various congenital anomalies. This prevalence is high compared the data previously reported in developing countries [8-10,21,22]. The high prevalence of congenital anomalies in the present study may be explained by the methodology employed by looking for congenital anomalies among young infants admitted to a tertiary referral hospital. Most young infants with congenital anomalies from regions surrounding Lake Victoria are referred to Bugando Medical Centre, as this is the only referral and consultant hospital capable of performing specialized investigation as well as pediatric surgical care. In addition, the high prevalence observed in this study could also be attributed to the type of classification used in this study (ICD10) which does not differentiate minor and major anomalies. A high prevalence of congenital anomalies of about 13% has also been reported among neonates admitted in neonatal intensive care units in low income countries [1]. Studies carried out among deliveries in tertiary hospitals have reported lower prevalence ranging between 1% and 7% [8-10,21,22].

In the current study, congenital anomalies affecting the CNS were the most common. This could be due to the fact that the surgical management of these malformations needs qualified personnel and special devices, such as ventricular-peritoneal shunt tubes, only available at BMC. Similar studies in Africa have reported a similar trend [23,24]. Periconceptual multiple vitamin supplements containing folic acid have been reported to reduce the incidence of neural tube defects and orofacial clefts [25-28]. Our results show that only 15% of the mothers of young infants had used folic acid during the first trimester of pregnancy and that non use folic acid was significantly associated with congenital anomalies. Due to the study design, the results however, were likely affected by many factors, including recall bias.. Our findings are similar to what was reported in Kenya and India where CNS, musculoskeletal system and gastrointestinal tract were the most affected systems [5,8]. Similar studies elsewhere have reported that the musculoskeletal and gastrointestinal systems are the body systems most commonly affected [9,18]. Differences in the pattern of body systems affected by congenital anomalies among different populations could reflect aetiological factors, such as genetic and environmental factors [2].

A significant association between congenital anomalies and the lack or peri-conceptional use of folic acid was found in this study. Folic acid is known to be necessary for the growth and smooth function of human cells, as it is crucial for the biosynthesis and methylation of deoxyribonucleic acid (DNA) and ribonucleic acid (RNA) [25]. This is important for cell division, differentiation and regulation of gene expression, especially at a time of rapid cell division like during embryogenesis [25]. Folic acid is crucial for a normal brain and spinal cord development during the first 4 weeks of gestation [2]. Several studies have shown that folic acid reduces the occurrence of some congenital anomalies e.g. neural tube defects, oro-facial clefts, limb reduction defects, congenital heart defects, urinary system defects and omphalocele [26,27]. The low usage of folic acid during the first trimester of pregnancy in this study could explain the higher proportion of neural tube defects and omphaloceles observed. The association between a low usage of folic acid during pregnancy and the occurrence of congenital anomalies has also been reported elsewhere [28].

The factors found to be significantly associated with congenital anomalies included an inadequate attendance to antenatal clinic, a maternal age above 35 years and a birth order of above 4 children. Antenatal clinic visits are an important part of prenatal care. During those visits, health education is usually given on various issues including adequate nutrition, avoidance of teratogens and maternal infections. Multiple vitamin supplements containing folic acid are also distributed during the clinic sessions [29]. The antenatal visits therefore aim at ensuring a normal pregnancy with the delivery of a healthy baby from a healthy mother. Few (≤3) or no prenatal clinic visits have previously been associated with the occurrence of congenital anomalies [30].

Gametogenesis in females begins before birth and the first meiotic division is usually completed shortly before ovulation. Sometimes, the first meiotic division may take a long time to be completed, e.g. up to 45 years. In such circumstances, the chances for meiotic errors from exposure to teratogens are very high because the oocyte spends a longer time in a dividing stage (prophase stage). The risk for congenital anomalies from chromosomal abnormalities as maternal age increases (especially above 35 years) is expected to be high [8,10,12]. An association between a high birth order and the occurrence of congenital anomalies was previously reported [2,21]. This link was attributed to the increased rate of mutation after the 3^rd^ gravid compared to the first and second gravid, as well as a higher maternal age [2,31].

A singleton pregnancy, a birth weight of 2.5 kg and above, a birth order of 4 and above and female sex were also found to be associated with congenital anomalies. We were unable to explain these findings due to the design of our study.

Our findings indicate that maternal smoking (passive smoking), alcohol consumption and a family history of congenital anomalies were not associated with congenital anomalies. Maternal cigarette smoking and alcohol consumption have previously been reported as risk factors for the occurrence of congenital anomalies including orofacial clefts and congenital heart diseases [15,32-34]. Cigarette smoking and alcohol intake are not common among Tanzanian women due to cultural norms. In addition, under ascertainment bias could have also affected our results due to non-reporting of family history because of shame, etc. A family history of birth defects has been associated with an increased risk of having another children with congenital anomalies, with a recurrence rate ranging between 2 and 5% and 1% for neural tube defects and Down syndrome respectively [35].

The significance of the association between congenital anomalies and various factors in this study should be interpreted with care. As a matter of fact, the information on associated factors was obtained through interviews and is likely to be affected by recall bias of former exposure to risk variables as well as denial from parents regarding the exposure to some of the factors due to fact occurrence of congenital anomalies is a delicate issue to most families. In addition, our results of associated factors are also likely to be influenced by under-ascertainment and use of BMC as the only data source.

## Conclusion

The high prevalence of congenital anomalies observed in this study calls for a special attention on this problem. We recommend that the hospital should mobilize more resources for an optimal and timely management of patients with congenital anomalies. Large community-based studies should be conducted in Tanzania to determine the prevalence of congenital anomalies among the newborns and their associated factors. The results could shed light on the pattern as well as the various risk factors for congenital anomalies.

In this study, the proportion of women taking folic acid supplements during early pregnancy was very low. Even though, due to its design, our study could not establish a causal relationship between non-use of folic acid and the occurrence of congenital anomalies, efforts should be made to ensure that more women use folic acid during the periconceptional period since there is ample documentation about its association with congenital anomalies Similarly, pregnancies and deliveries at an advanced age should be discouraged as they have been associated in various studies with the occurrence of congenital anomalies. Large community-based studies in different geographical, environmental and socio-economic settings should be conducted in Tanzania to determine the prevalence of congenital anomalies and their associated factors.

## Competing interests

There were no financial or non-financial competing interests.

## Authors’ contributions

**FM** participated in the design of the study, data collection and analysis, as well as helped to draft the manuscript; **AZ** participated in the design of the study and performed some of the radiological investigations on young infants; **PC** participated in the design and helped draft the manuscript; **BRK** participated in statistical analysis and helped draft the manuscript; **MM** participated in the design of the study, data analysis and drafted the manuscript. All authors read and approved the final manuscript.
